# Electrophysiological correlates of ketamine and esketamine in humans: a systematic review of resting EEG findings

**DOI:** 10.1192/j.eurpsy.2026.11627

**Published:** 2026-07-31

**Authors:** L. Mana, R. Lisbino, P. Pezzella, A. Perrottelli, F. F. Marzocchi, N. Sansone, A. Mucci, S. Galderisi

**Affiliations:** Psychiatry, University of Campania Luigi Vanvitelli, Naples, Italy

## Abstract

**Introduction:**

Ketamine and esketamine exert rapid antidepressant and dissociative effects, and the growing clinical use of intranasal esketamine for depression opens the possibility of large-scale electroencephalography (EEG) studies to explore their mechanisms through a non-invasive approach. However, understanding the neurophysiological basis of these effects requires a clear synthesis of existing evidence on how ketamine modulates human cortical dynamics. Yet, despite a growing body of research, no comprehensive review has systematically investigated the electrophysiological correlates of ketamine’s effects in humans.

**Objectives:**

This review aims to fill these gaps by systematically characterizing EEG alterations induced by ketamine and esketamine in humans, with a particular focus on how electrophysiological changes unfold over different temporal scales, from the immediate neuropharmacological phase to potential longer-term adaptations.

**Methods:**

To this aim, we will conduct a systematic review of original EEG studies in humans published over the past 30 years (1995–2025) investigating the effects of ketamine or esketamine in both healthy volunteers and clinical populations. EEG alterations will be categorized across multiple domains, with particular focus on studies reporting associations with clinical outcomes. Findings will be organized according to temporal dynamics—acute (<1 h), subacute (1–24 h), and longitudinal (≥24 h, including follow-up)—to capture both immediate and delayed effects.

**Results:**

Integrating diverse EEG measures within different temporal scales, this review seeks to identify distinct patterns of cortical modulation across acute and longer-term windows, revealing a structured progression of changes in frequency bands and functional connectivity following ketamine. Emerging evidence on intranasal esketamine suggests overlapping electrophysiological profiles.

**Conclusions:**

This systematic review will provide the first integrated overview of the temporal dynamics of EEG alterations induced by ketamine and esketamine in humans, offering a coherent model to interpret how electrophysiological dynamics relate to the onset of dissociative and early antidepressant effects. These insights aims to address gaps left by previous reviews and might support the design of future clinical EEG studies of esketamine induced therapeutic effects.

**Disclosure of Interest:**

None Declared

